# Simultaneous Whole-Cell Recording and Calcium Imaging Does Not Reveal Electrically Coupled Neurons in *Xenopus* Tadpoles

**DOI:** 10.1523/ENEURO.0073-26.2026

**Published:** 2026-05-14

**Authors:** Bella Xu-Ying, Maarten F. Zwart, Wen-Chang Li

**Affiliations:** ^1^School of Psychology and Neuroscience, University of St Andrews, St Andrews KY16 9JP, United Kingdom; ^2^Institute of Behavioural and Neural Sciences, University of St Andrews, United Kingdom; ^3^Centre of Biophotonics, University of St Andrews, United Kingdom

**Keywords:** calcium imaging, electrical coupling, gap junction, GCaMP, whole-cell recording

## Abstract

Neuronal populations connected by gap junctions can be revealed via dye coupling of small molecules like neurobiotin and Lucifer yellow. However, the extent of dye diffusion between neurons varies with connexin subtype, loading method, and neuromodulation. Due to the increasing availability of GCaMP transgenic animals, we explore the possibility of revealing gap junctional coupling using Ca^2+^ imaging in the motor system of *Xenopus laevis* tadpole of either sex. Reliable axo-axonal electrical coupling was previously found in excitatory descending interneurons (dINs) using paired recordings but not with neurobiotin dye coupling. Here, we made whole-cell patch–clamp recordings with Ca^2+^-supplemented intracellular solution to load Ca^2+^ into GCaMP6-expressing neurons, followed by Ca^2+^ imaging to detect potential Ca^2+^ diffusion across coupled neurons. Successful membrane breakthroughs led to transient fluorescence increases in the patched neuron. However, increasing the Ca^2+^ concentration promoted membrane resealing and rapid loss of whole-cell recordings. Regardless of recording duration, loading-triggered fluorescence only lasted up to 3 min, suggesting rapid Ca^2+^ clearance. Pharmacologically blocking sarcoplasmic/endoplasmic reticulum Ca^2+^-ATPases and plasma membrane Na^+^/Ca^2+^ exchangers did not prolong fluorescence, although sustained fluorescence was achieved with positive current injections. Counter to our expectations, fluorescence increases in Ca^2+^-loaded dINs did not spread to neighboring dINs. Robust intracellular Ca^2+^ regulation mechanisms, membrane resealing, and long dIN axons likely hindered intercellular Ca^2+^ diffusion. Therefore, this approach is not appropriate for revealing electrical coupling within this system.

Significance Statement

Gap junctions mediate electrical coupling but are only revealed by dye coupling in some neuronal populations. Using transgenic *Xenopus* GCaMP6s tadpoles, we attempted to reveal gap junction connections among a population of premotor interneurons, previously shown to be electrically coupled, by loading free Ca^2+^ via whole-cell recordings and imaging potential Ca^2+^ diffusion. Ca^2+^ loading increased fluorescence for up to 3 min in the recorded neuron but also promoted membrane resealing and loss of recordings. This fluorescence increase was prolonged by positive current injections, but not by pharmacologically blocking Ca^2+^-ATPases and Na^+^/Ca^2+^ exchangers. Counter to our expectations, fluorescence increases were not detected in surrounding neurons. Therefore, imaging Ca^2+^ diffusion is not a feasible approach to reveal gap junction connections in this system.

## Introduction

Gap junctions are essential in cell-to-cell communication. In epithelial cells, glia, and cardiac muscle cells, gap junctions are found ubiquitously and help form extensive cell networks (Severs, 1994; Orthmann-Murphy et al., 2008; Giepmans and van IJzendoorn, 2009). In contrast, neuronal gap junctions, or electrical synapses, are restricted to specific cell types and developmental stages (Söhl et al., 2005). These channels play important roles in activity synchronization within neural populations (Zhang et al., 2009), recurrent excitation (Liu et al., 2016), development (Cao et al., 2023), and cell survival (Belousov et al., 2017). Consequently, gap junction defects have been associated with various neurodegenerative diseases such as Alzheimer's and Parkinson's (Sánchez et al., 2020; Xing and Xu, 2021; Choudhury et al., 2022). The different gap junction expression patterns across neuron types require reliable and effective methods to determine the extent of electrical coupling.

Standard methods for revealing gap junction-connected cells include paired recordings and dye coupling. In paired recordings of coupled cells, applying current injections to one cell results in transjunctional voltage changes in the other (Bennett et al., 1963). Although highly sensitive, the technical challenges involved in performing paired recordings and its limitation to two cells per experiment reduce efficiency and throughput (Abbaci et al., 2008). In dye coupling experiments, small dyes or tracers like neurobiotin, Lucifer yellow, and biocytin (Liu et al., 2016) or serotonin (Szarka et al., 2023) can reveal coupled cells or networks by diffusing across gap junctions (Stephan et al., 2021). This is less technically challenging than paired recordings, but the extent of dye coupling hinges on several uncertainties. First, dye microinjections or scrape loading can cause cell damage, making the amount of dye loaded difficult to standardize (Abbaci et al., 2008). This damage can trigger neuromodulation and homeostatic mechanisms that affect gap junction permeability (Ziambaras et al., 1998; Bloomfield and Völgyi, 2009). Moreover, gap junctions have different dye permeabilities depending on their connexin composition. For example, Lucifer yellow can pass through Cx40, Cx43, and Cx45 gap junctions but not Cx30.2 (Rackauskas et al., 2007). These dependencies present challenges in studying electrical synapses, especially in cells with gap junctions of unknown connexin makeup.

In recent years, new genetically encoded methods have emerged, many of which use a “generator-reporter” system (Dong et al., 2018). Ion channels or pumps are expressed in “generator” cells and sensor proteins in coupled “reporter” cells. The experimenter can then trigger an inflow of ions into the “generator” (e.g., via optogenetics), and the ions’ diffusion across gap junctions is detected by the “reporter” (e.g., via fluorescence sensor proteins). These methods are feasible in *Drosophila melanogaster* (Wu et al., 2019) and cell cultures (Kang and Baker, 2016) where sophisticated genetic toolkits are available. However, for many model systems in which similar tools are lacking, such as *Xenopus laevis*, studying gap junctions is limited to conventional techniques like paired recordings and dye coupling. Previously, paired recordings from the swimming motor circuits in *Xenopus* tadpoles revealed electrical coupling among descending interneurons (dINs) through potential axo-axonal gap junctions (Li et al., 2009; Zhang et al., 2009). Random paired recordings showed that 90% of dIN pairs (55/61) were electrically coupled, with coupling coefficients (3–17%) decreasing with intra-pair soma distance (13–189 µm). Despite this extensive coupling among dINs, neurobiotin rarely diffused beyond the injected cell, leading to an absence of dye coupling in 94% of dINs (78/83; Li et al., 2009). Due to these inconsistencies across methods, our understanding of dIN coupling at the population level remains limited (e.g., how many dINs are coupled to each other at different longitudinal positions along the caudal hindbrain and spinal cord), hindering functional understanding of their role in locomotion.

We attempted to explore an alternative method for revealing electrical coupling among *Xenopus* tadpole dINs: using whole-cell electrodes to load Ca^2+^ into GCaMP-expressing neurons and Ca^2+^ imaging to detect transjunctional Ca^2+^ diffusion to potentially coupled cells. Ca^2+^ ions (40 Da) are smaller than neurobiotin (286 Da; Mills and Massey, 1998) and should diffuse more readily through gap junctions. Moreover, we used a common pan-neuronal GCaMP6s line available to many model systems, removing the need for complex genetic tool development. We loaded Ca^2+^ into single cells and monitored changes in GCaMP6s fluorescence upon membrane breakthrough. First, we showed that loading Ca^2+^ produced a rapid fluorescence increase in the recorded neuron with an average decay halftime of 103 s. Raising the Ca^2+^ concentration and applying pharmacological blockers of Ca^2+^ pumps, which normally remove cytoplasmic Ca^2+^, did not prolong the fluorescent signal. Instead, loading high Ca^2+^ concentrations promoted resealing of the cell membrane within the electrode and rapid loss of whole-cell recordings. Although positive current injections could sustain fluorescence increases within the loaded cell, loading dINs with Ca^2+^ did not lead to detectable fluorescence increases in neighboring dINs. We therefore conclude that this is not a viable method for revealing electrical coupling between *Xenopus* tadpole dINs.

## Materials and Methods

### Animals

Pairs of adult *Xenopus laevis* frogs of opposite sex were induced to breed via injections of 1,000 U/ml human chorionic gonadotropin hormone (Sigma-Aldrich). Fertilized embryos were collected and raised at temperatures between 16 and 22°C to stagger developmental rates. Stage 37/38 tadpoles, the sex of which cannot be determined before metamorphosis, were used in all experiments. All procedures were approved by the local Animal Welfare Ethics Committee and complied with UK Home Office regulations (license number PP2896979).

### Dissection

Tadpoles were anesthetized for 10–20 s in 0.1% 3-aminobenzoic acid ester (MS-222, Sigma-Aldrich) to allow opening of the dorsal fin and then immobilized for 25 min with 13 µM α-bungarotoxin (Sigma-Aldrich). Immobilized tadpoles were pinned to a rotatable Sylgard dissection dish filled with extracellular saline (in mM: 115 NaCl, 10 HEPES, 3 KCl, 2 CaCl_2_, 1 MgCl_2_, 2.4 NaHCO_3_), adjusted with 5 M NaOH to pH 7.4, with an osmolarity of ∼257 Osm/L. Most of the yolk sac and the rostral trunk skin covering both sides of myotome blocks were dissected out. The skin covering the hindbrain was removed, and a slit was made dorsally along the roof of the fourth ventricle and rostral spinal cord to open respective neurocoeles. Ependymal cells lining the neurocoele were removed with a fine dissection needle to expose hindbrain and spinal cord somas. The left side of the caudal hindbrain and rostral spinal cord was removed (hemicord) to improve visibility using the transmitted light on an E600FN microscope (Nikon).

### Whole-cell patch–clamp recordings and Ca^2+^ loading

After dissection, tadpoles were transferred to a rotatable recording dish, and whole-cell patch–clamp recordings in current-clamp mode were made following established methods (Li, 2021). Electrodes were pulled with a P-87 pipette puller (Sutter Instrument) with DC resistances between 10 and 20 MΩ. The standard intracellular solution contained the following (in mM): 100 K-gluconate, 2 MgCl_2_, 10 EGTA, 10 HEPES, 3 Na_2_ATP, 0.5 NaGTP, and 3.1 neurobiotin, adjusted with KOH to pH 7.3, with an osmolarity of ∼239 Osm/L. For Ca^2+^ loading experiments, CaCl_2_ was added and Ca^2+^ chelators EGTA, K-gluconate and Na_2_ATP were replaced with K-CH_3_SO_3_ and MgATP (Woehler et al., 2014) for final concentrations of the following (in mM): 107 K-CH_3_SO_3_, 1 MgCl_2_, 2 CaCl_2_, 10 HEPES, 3 MgATP, 0.5 NaGTP, and 3.1 neurobiotin, adjusted with KOH to pH 7.3, with an osmolarity of 243 Osm/L. In the 10 mM Ca^2+^ loading experiments, the intracellular solution with 10 mM CaCl_2_ had an osmolarity of 267 Osm/L. We therefore increased the extracellular saline osmolarity accordingly by raising NaCl to 127 mM for an osmolarity of ∼281 Osm/L. Epifluorescence Ca^2+^ imaging was performed with an Andor Neo5.5 CMOS camera and Andor Solis software (Oxford Instruments). Blue light was supplied via a 480 nm LED array module on a pE-1 system (CoolLED). LED lighting and image capture were synchronized through TTL pulses configured through the Signal software (Cambridge Electronic Design). Intracellular signals were amplified with a Multiclamp 700B amplifier (Molecular Devices). Fictive swimming was monitored by recording motor nerve activity from myotome clefts with a glass suction electrode, amplified with a four-channel extracellular amplifier (AM Systems), and digitized with a Power1401 Digitizer (Cambridge Electronic Design). All electrophysiological recordings were acquired at a 10 kHz sampling rate using Signal.

The seal between the patch-clamp electrode and the neuronal membrane was monitored with 2 ms, 50 pA test current pulses at 1 Hz in current-clamp mode. Once a giga-ohm seal was formed, large zapping currents (five 200 Hz 2 ms 5 nA pulses) were applied to break the cell membrane inside the whole-cell recording electrode. We used the following criteria to assess successful membrane breakthroughs and the attainment of proper whole-cell recordings: an immediate drop in voltage to the neuronal resting membrane potential, an instant decrease in voltage responses to test current pulses, a reduction in 50 Hz mains AC noise, the appearance of spontaneous synaptic potentials, and the presence of full-size action potentials overshooting to +20 mV or above in response to current injections or during fictive swimming. During membrane resealing, these features disappeared, and the whole-cell recording was lost. During more stable recordings, positive current injections were used to drive extra Ca^2+^ into the recorded neuron. Current steps were applied at 1 Hz with amplitudes and durations ranging from 100 to 600 pA and 300 to 600 ms for 1–3 min injection periods, depending on recording stability.

### Loose-patch extracellular recordings

For loose-patch recordings, gentle suction was applied to the neuron without breaking the cell membrane and spiking was recorded through evoked fictive swimming. dINs and non-dINs were classified by differences in their extracellular spike shapes (Soffe et al., 2009); dINs have longer peak-to-trough durations and smaller trough amplitudes (normalized to total peak-to-trough amplitude). One target dIN in each preparation was then whole-cell recorded to load 2 mM Ca^2+^. Target dIN identities were confirmed by neurobiotin staining following established methods (Li et al., 2001).

### Fluorescence analysis

Ca^2+^ imaging videos were exported to Fiji (Schindelin et al., 2012). XY drift was corrected with the Correct 3D Drift plugin (Parslow et al., 2014), and regions of interest (ROIs) were drawn around the patched neurons. A large void region outside of the neural tissue was used to calculate background fluorescence. The background fluorescence was then subtracted from the neuron ROI’s mean area pixel intensity to obtain fluorescence (*F*) values. The baseline fluorescence (*F*_0_) was calculated as the 10th percentile *F* value 5 s before membrane breakthrough. Δ*F*/*F* as a percentage was then calculated as follows:

$$\Delta F/F\;=\frac{\left(F-F_{0}\right)}{F_{0}}\times 100.$$

The following parameters were used to assess fluorescence dynamics after breakthrough: fluorescence increase was calculated as the maximum fluorescence after membrane breakthrough, rate of increase was the rate at which fluorescence reached its maximum value from breakthrough, and halftime was the time taken for fluorescence to drop to 50% of the maximum value.

### Pharmacology

In some experiments, we added 1 µM thapsigargin (Tocris Bioscience), 10 µM SEA 0400 (Sigma-Aldrich), 0.31 µM tetrodotoxin (TTX; Sigma-Aldrich), and 150 µM Cd^2+^ (Sigma-Aldrich) to the extracellular saline to block sarcoplasmic/endoplasmic reticulum Ca^2+^-ATPases (SERCAs; Lytton et al., 1991), plasma membrane Na^+^/Ca^2+^ exchanger NCX1.1 (Bouchard et al., 2004; Lee et al., 2004), voltage-gated Na^+^ channels (Bane et al., 2014), and voltage-gated Ca^2+^ channels (Hinkle et al., 1987), respectively. This was done to facilitate Ca^2+^ diffusion to coupled cells and isolate loading-triggered Ca^2+^ increases from other physiological processes affecting cytoplasmic Ca^2+^. Thapsigargin inhibits all human SERCA family proteins (SERCA1, SERCA2a, SERCA2b, SERCA3) with equal potency (Lytton et al., 1991). The *Xenopus* SERCA2 has 92% percentage identity to human SERCA2, and the *Xenopus serca2/atp2a2* gene is expressed in neural tissue at stage 37/38 (Pegoraro et al., 2011). SEA 0400 blocks NCX1.1, a human Na^+^/Ca^2+^ exchanger (Bouchard et al., 2004; Lee et al., 2004). We performed a BLAST analysis which revealed a *Xenopus* ortholog of NCX1.1, slc8a1.S, with high percentage identity (84.05%), which is expressed in tadpole neural tissue (Raciti et al., 2008).

### Statistical analysis

Shapiro–Wilk normality tests and Levene's tests of equality of variances were used to select the appropriate statistical tests. For normally distributed data, Student’s *t* tests were used. For non-normally distributed data, Mann–Whitney *U* tests and Kruskal–Wallis tests were used. Welch corrections for unequal variances and Holm corrections for multiple comparisons were used, when applicable. A Pearson’s correlation was used for regression analysis. All in-text descriptive statistics depict mean ± 1SD.

## Results

### Loading Ca^2+^ into neurons causes transient GCaMP6s fluorescence increases

**Figure 1.**
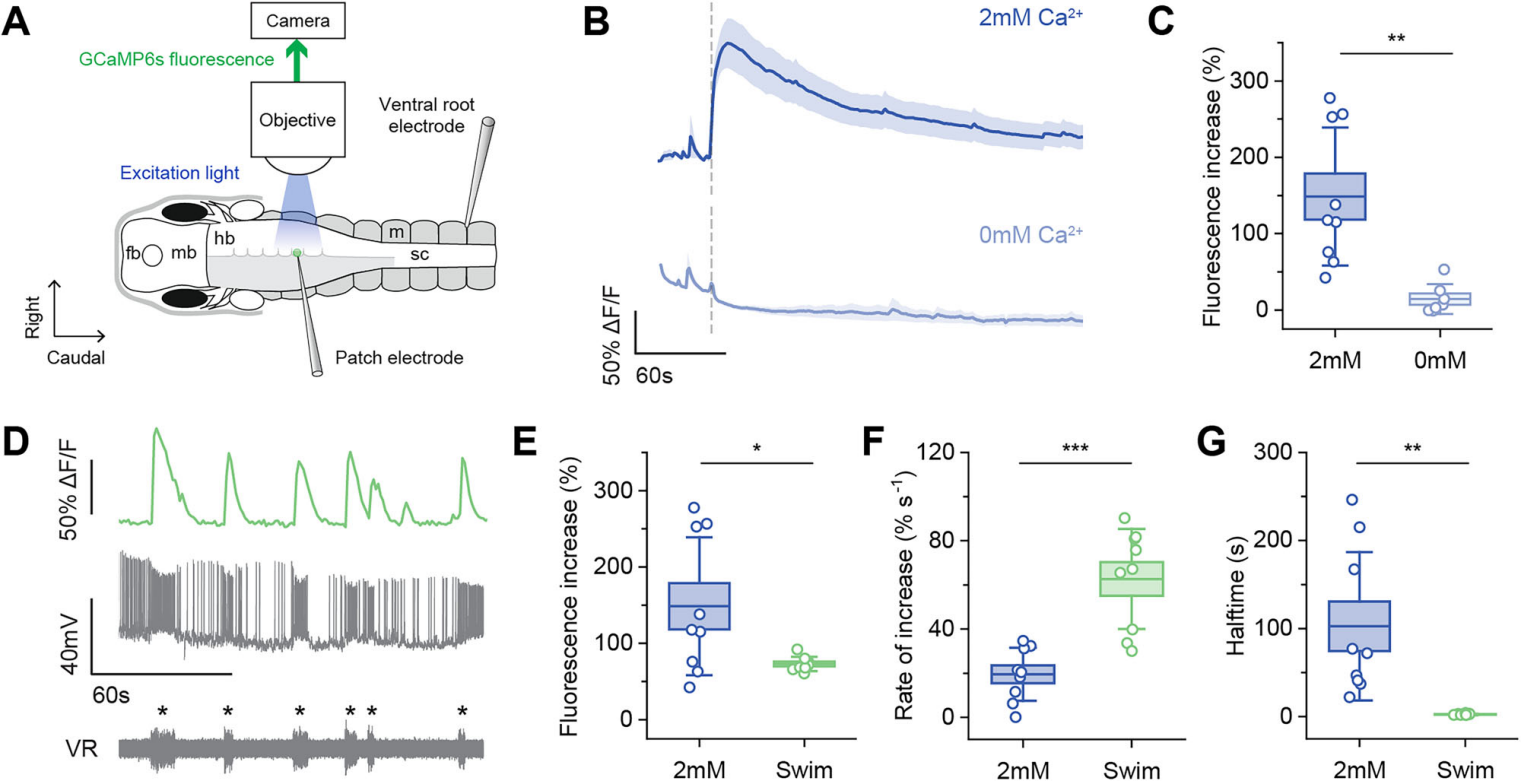
Loading Ca^2+^ into neurons through patch-clamp electrodes transiently increases GCaMP6s fluorescence. ***A***, Dorsal view of a hemi-hindbrain tadpole CNS and diagram of the Ca^2+^ loading setup. The patch electrode is filled with intracellular solution containing 2 or 0 mM Ca^2+^. The ventral root electrode monitors fictive swimming activity. Fb, forebrain; mb, midbrain; hb, hindbrain; sc, spinal cord; m, myotome. ***B***, Averaged (mean ± SEM) GCaMP6s fluorescence of the recorded neuron upon membrane breakthrough (vertical dashed line) with intracellular solution containing 2 and 0 mM Ca^2+^. 2 mM: *n* = 9, 0 mM: *n* = 7. ***C***, Maximum fluorescence increase reached after 2 mM or 0 mM Ca^2+^ loading. Boxes: mean ± SEM, whiskers: ±SD; 2 mM: *n* = 9, 0 mM: *n* = 7; Student’s *t* test with Welch corrections for unequal variances, **p < 0.01. ***D***, GCaMP6s fluorescence and whole-cell recording of a hindbrain neuron with simultaneous ventral root (VR) recording of six spontaneous fictive swimming episodes (marked with asterisks). ***E***–***G***, Comparing fluorescence dynamics between Ca^2+^ loading and fictive swimming: maximum fluorescence increase, rate of increase, and decay halftime. Boxes, mean ± SEM; whiskers, ±SD; 2mM, *n* = 9; swim, *n* = 9; Student’s *t* tests for normally distributed data (fluorescence increase, rate of increase), Mann–Whitney *U* test for non-normally distributed data (halftime); *p < 0.05; **p < 0.01; ***p < 0.001.

We first loaded Ca^2+^ into random hindbrain and spinal neurons using whole-cell electrodes to see if this could trigger GCaMP6s fluorescence increases. We added 2 mM Ca^2+^ to the intracellular solution and performed whole-cell recordings with simultaneous Ca^2+^ imaging to capture fluorescence changes at the time of membrane breakthrough (Fig. 1*A*). We found significant increases in fluorescence upon breakthrough (148.6 ± 90.3%; *n* = 9) compared with standard intracellular solution with 0 mM free Ca^2+^ (14.4 ± 19.4%; *n* = 7; *p* < 0.01; Fig. 1*B*,*C*; Movie 1). Next, to see if these dynamics differed to physiological single-cell activity, we compared fluorescence increases induced by Ca^2+^ loading and during fictive swimming (Fig. 1*D*). Fictive swimming bouts produced fluorescence peaks of smaller amplitude (swim, 73.1 ± 9.3%; *n* = 9; Ca^2+^, 148.6 ± 90.3%; *n* = 9; *p* < 0.05; Fig. 1*E*) but faster rates of increase compared with Ca^2+^ loading (swim, 62.7 ± 22.6% s^−1^; *n* = 9; Ca^2+^, 19.5 ± 12.0% s^−1^; *n* = 9; *p* < 0.001; Fig. 1*F*). Ca^2+^ loading-triggered fluorescence also had considerably longer halftime than fictive swimming (swim, 2.7 ± 0.7 s; *n* = 9; Ca^2+^, 102.7 ± 84.1 s; *n* = 9; *p* < 0.01; Fig. 1*G*).

**Movie 1.** Calcium imaging of one neuron loaded with 2 mM Ca^2+^ (left) and another with 0 Ca^2+^ (right). Videos are sped up 15 times. [View online]

### Increasing intracellular Ca^2+^ concentration promotes membrane resealing

**Figure 2.**
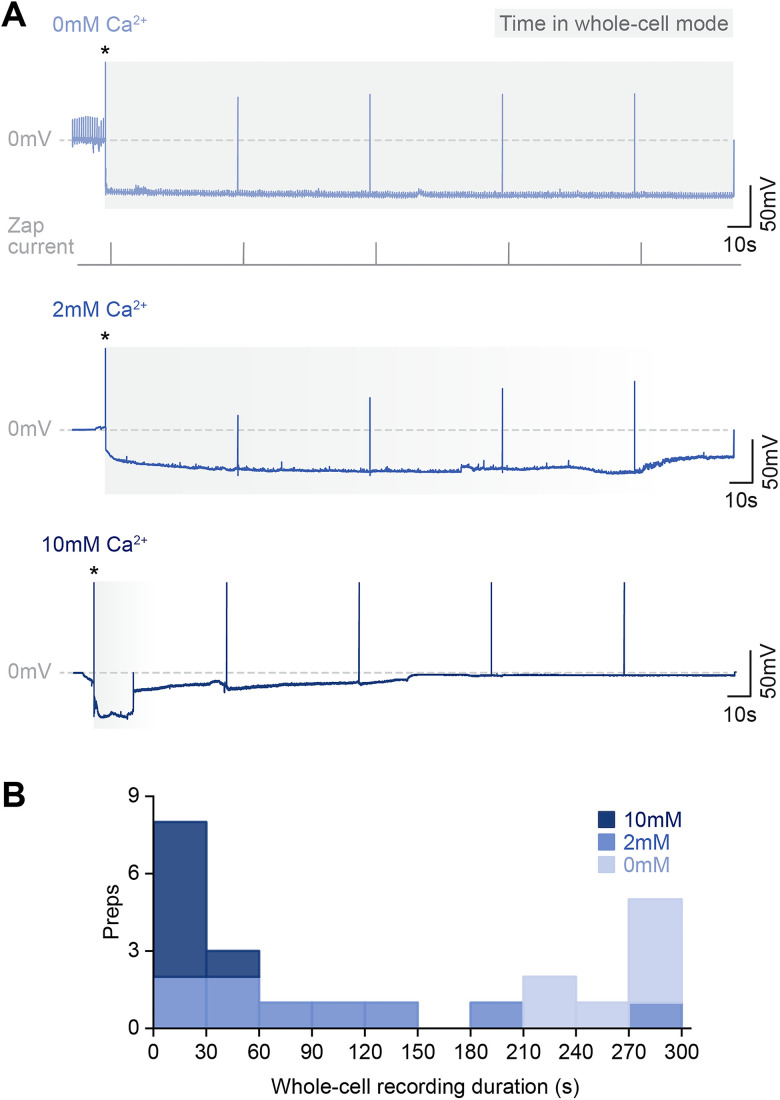
Increasing intracellular Ca^2+^ concentration promotes membrane resealing and loss of whole-cell recordings. ***A***, Whole-cell recording traces with 0, 2, and 10 mM Ca^2+^ showing the progression of membrane resealing with increased Ca^2+^ concentration, e.g., drift in membrane potential in 2 and 10 mM traces. Zap current is applied for membrane breakthrough to enter whole-cell mode (*) and then periodically to monitor recording stability. ***B***, Stacked histogram showing whole-cell recording durations for 0, 2, and 10 mM Ca^2+^. Duration starts from membrane breakthrough (marked by asterisks in ***A***) and ends when physiological features characteristic of stable whole-cell recordings are lost (see Materials and Methods). 0 mM, *n* = 7; 2 mM, *n* = 9; 10 mM, *n* = 7.

To maximize Ca^2+^ diffusion to potentially coupled neurons, we increased the Ca^2+^ concentration to 10 mM. However, whole-cell recordings with 10 mM Ca^2+^ could not be sustained as signs of membrane resealing appeared which led to the loss of whole-cell recordings (see Materials and Methods; Fig. 2*A*). Upon reexamination, our 2 mM recordings also displayed indications of resealing (Fig. 2*A*). Whereas 100% of our 0 mM recordings could be held in whole-cell mode for over 3 min, 78% of 2 mM recordings showed resealing within 3 min, and 100% of 10 mM recordings showed resealing within 1 min of membrane breakthrough (Fig. 2*B*). Recordings with 10 mM Ca^2+^ were significantly shorter than those for 0 and 2 mM (independent-sample Kruskal–Wallis test*, p* < 0.05 and *p* < 0.001, respectively).

### Blocking Ca^2+^ exchangers and pumps does not prolong fluorescence

**Figure 3.**
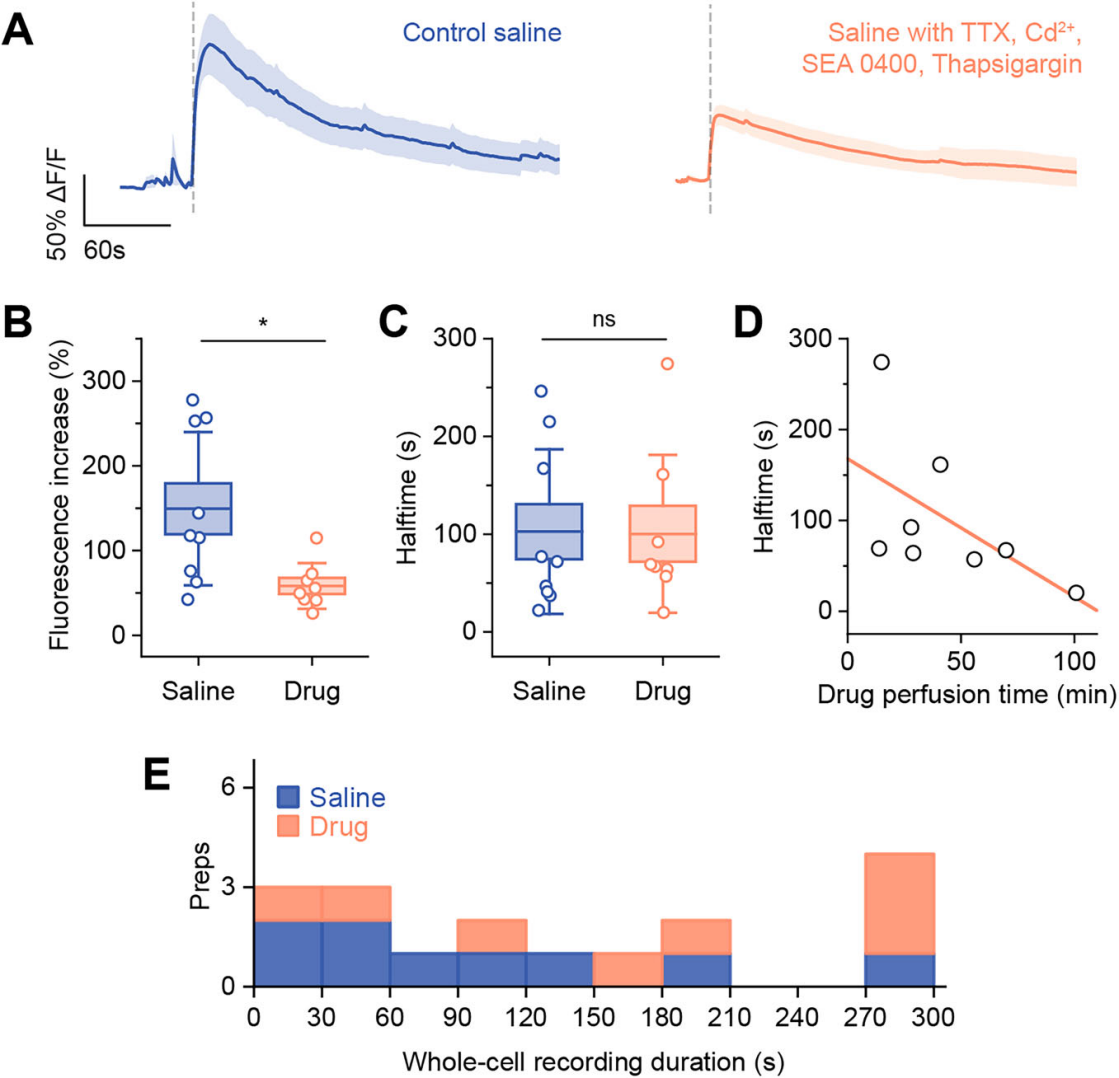
Ca^2+^ exchanger and pump blockers do not prolong fluorescence increases produced by 2 mM Ca^2+^ loading. ***A***, Averaged (mean ± SEM) fluorescence upon membrane breakthrough (vertical dashed lines) in saline versus drug perfusion. ***B***,***C***, Comparing maximum fluorescence increase and decay halftime in saline versus drug perfusion. Boxes, mean ± SEM; whiskers, ±SD; saline, *n* = 9; drugs, *n* = 8; Mann–Whitney *U* tests, *p < 0.05. ***D***, Scatterplot showing drug perfusion time before membrane breakthrough does not affect fluorescence halftime. Pearson’s correlation, *r*^2^ = 0.20; *p* = 0.15; *n* = 8. ***E***, Stacked histogram showing whole-cell recording durations for saline versus drug perfusion. Duration starts from membrane breakthrough (vertical dashed lines in ***A***) and ends when physiological features characteristic of stable whole-cell recordings are lost (see Materials and Methods). Saline, *n* = 9; drugs, *n* = 8.

Since stable whole-cell recordings could not be sustained with 10 mM Ca^2+^ intracellular solution, we applied blockers of Ca^2+^ exchangers and pumps to see if we could prolong fluorescence increases during 2 mM Ca^2+^ loading. Specifically, we used thapsigargin to block SERCA2s and SEA 0400 to block plasma membrane Na^+^/Ca^2+^ exchanger NCX1.1. In addition, we added TTX and Cd^2+^ to block voltage-gated Na^+^ and Ca^2+^ channels to remove activity-dependent Ca^2+^ influx and ensure fluorescence changes occurred only due to Ca^2+^ loading. Compared with our previous 2 mM Ca^2+^ loading experiments in physiological saline, the presence of blockers lowered maximum fluorescence increase (saline, 148.6 ± 90.3%; *n* = 9; drugs, 69.2 ± 28.7%; *n* = 8; *p* = 0.046; Fig. 3*A,B*) but did not affect fluorescence decay halftime (saline, 102.7 ± 84.1 s; *n* = 9; drugs, 100.5 ± 80.8 s; *n* = 8; *p* = 1.0; Fig. 3*C*). Moreover, the duration of drug application had no effect on decay halftime (*r*^2^ = 0.20; *n* = 8; *p* = 0.15; Fig. 3*D*). Recordings were also similar in duration to those in control saline (independent-sample Kruskal–Wallis test*, p* = 0.2; Fig. 3*E*).

### Ca^2+^ loaded into dINs does not spread to other dINs

**Figure 4.**
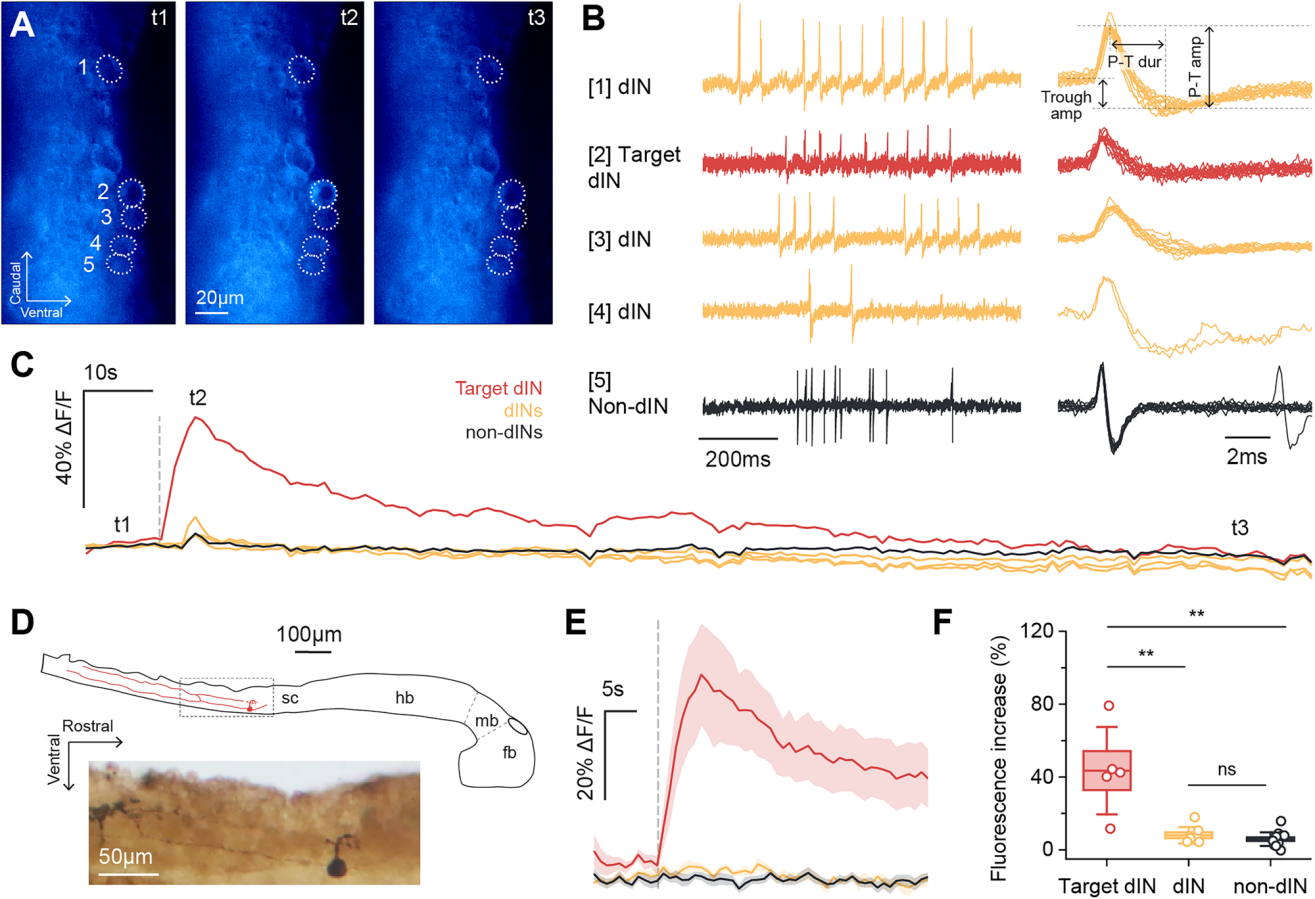
Ca^2+^ loading into target dINs does not lead to detectable fluorescence increases in other dINs and non-dINs. ***A***, Fluorescence images of neurons (dashed circles) at timepoints t1–t3 in the fluorescence trace in ***C***. Neurons 1–4 are dINs and Neuron 5 is a non-dIN based on their extracellular spike shapes in ***B***. Neuron 2 was recorded in whole-cell mode and loaded with 2 mM Ca^2+^. ***B***, Left, Loose-patch recordings of the five neurons in ***A*** during spontaneous swimming. Right, Superimposed extracellular spikes of each neuron and spike shape parameters measured. P-T dur, peak–trough duration; Trough amp, trough amplitude; P-T amp, peak–trough amplitude. ***C***, Fluorescence traces for the five neurons in ***A*** after membrane breakthrough in Neuron 2 over a 3 min period. Minimal fluorescence increases in other dINs and non-dINs at t2 are likely due to signal bleed-through from epifluorescence microscopy. ***D***, Top, Drawing of a whole-cell recorded dIN and its descending axon from neurobiotin morphological staining. Bottom, Photograph of section in gray dashed box. Fb, forebrain; mb, midbrain; hb, hindbrain; sc, spinal cord. ***E***, Averaged (mean ± SEM) fluorescence within 1 min of membrane breakthrough for each neuron group. ***F***, Comparing maximum fluorescence increases across neuron groups after target dIN breakthrough. Boxes, mean ± SEM; whiskers, ±SD; target dINs, *n* = 5; dINs, *n* = 8; non-dINs, *n* = 14; Kruskal–Wallis test with Mann–Whitney *U* post hocs, **p < 0.01.

Finally, we loaded Ca^2+^ into dINs and monitored if it could spread to neighboring dINs and increase their fluorescence. All neurons within the imaging field of view were first recorded in loose-patch mode to observe their spiking during swimming (Fig. 4*B*). Previous work found dIN spikes had longer spike durations and smaller trough amplitudes than non-dINs (Soffe, Roberts and Li, 2009). In 27 neurons across five preparations, we identified 13 dINs and 14 non-dINs based on these criteria. Similar to previous findings, dIN peak–trough duration was longer (dINs, 3.3 ± 1.4 ms; *n* *=* 13; non-dINs, 0.8 ± 0.3 ms; *n* *=* 14; Mann–Whitney *U* test, *p* *<* 0.001) and trough amplitude was smaller (dINs, 0.3 ± 0.08; *n* *=* 13; non-dINs, 0.5 ± 0.07; *n* *=* 14; Mann–Whitney *U* test, *p* *<* 0.001) compared with non-dINs. Within each preparation, one target dIN was recorded in whole-cell mode and loaded with Ca^2+^, while other dINs and non-dINs (controls) in the field of view were monitored for fluorescence changes (Fig. 4*A*–*C*). dINs recorded in whole-cell mode were confirmed as dINs via morphological staining of their descending axons (Fig. 4*D*). We compared the fluorescence signals of the target dIN with surrounding loose-patched neurons (Fig. 4*E*). Target dINs showed increases in fluorescence after breakthrough (43.5 ± 24.0%; *n* = 5), which was significantly greater than surrounding dINs (8.0 ± 4.5%; *n* = 8; *p* < 0.01) and non-dINs (5.9 ± 3.8%; *n* = 14; *p* < 0.01; Fig. 4*F*). The fluorescence of these surrounding neurons did not differ from each other (*p* = 0.3) and remained unchanged even after 3 min (Fig. 4*C*).

**Figure 5.**
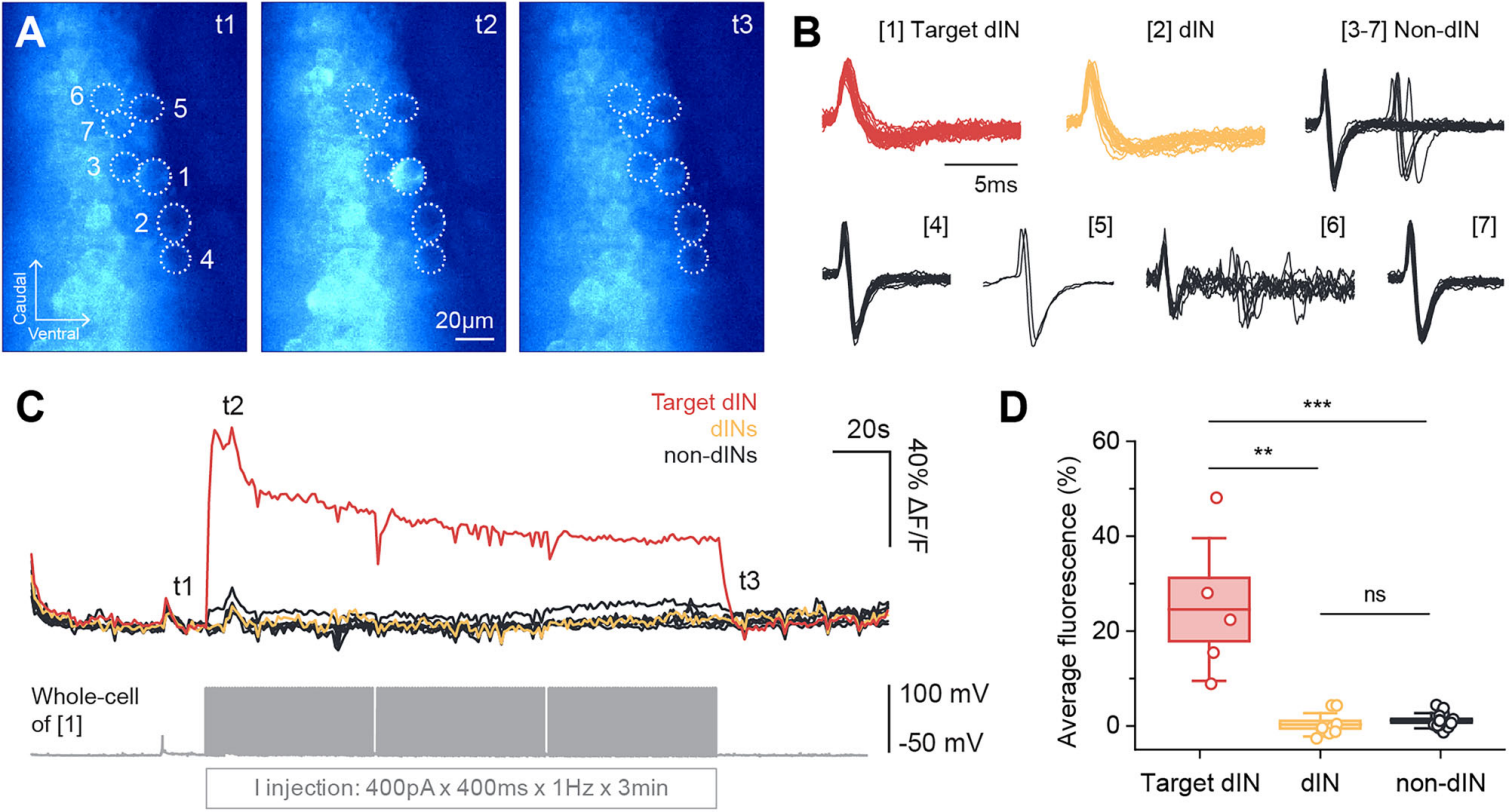
Extra Ca^2+^ loading through positive current injections into target dINs does not lead to detectable fluorescence increases in other dINs and non-dINs. ***A***, Fluorescence images of neurons (dashed circles) at timepoints t1–t3 in the fluorescence trace in ***C***. Neurons 1–2 are dINs and Neurons 3–7 are non-dINs based on their extracellular spike shapes in ***B***. Neuron 1 was recorded in whole-cell mode and loaded with 2 mM Ca^2+^. ***B***, Superimposed extracellular spikes of the neurons in ***A*** during swimming. ***C***, Fluorescence traces for the seven neurons in ***A*** during 3 min of positive current injection into the loaded dIN (Neuron 1). ***D***, Comparing average fluorescence during the current injection period across neuron groups. Boxes, mean ± SEM; whiskers, ±SD; target dINs, *n* = 5; dINs, *n* = 9; non-dINs, *n* = 17; Kruskal–Wallis test with Mann–Whitney *U* post hocs, **p < 0.01; ***p < 0.001.

In five different preparations, the whole-cell recordings were stable enough for us to inject positive current steps to drive extra Ca^2+^ into the dINs after the initial Ca^2+^ signal had faded. Current injections increased fluorescence in the patched dIN to its peak within a few seconds, followed by a plateau that lasted until the end of the current injection period (Fig. 5*A*–*C*). However, this did not produce fluorescence increases in neighboring neurons. The average fluorescence of target dINs across the current injection period (24.5 ± 15.0%; *n* = 5) was significantly higher than surrounding dINs (0.3 ± 2.5%; *n* = 9; *p* < 0.01) and non-dINs (1.1 ± 1.6%; *n* = 17; *p* < 0.001; Fig. 5*D*). As before, the fluorescence of surrounding dINs and non-dINs did not differ (*p* = 0.1; Fig. 5*C*).

## Discussion

In this study, we explored Ca^2+^ loading via whole-cell recording electrodes as a potential method for revealing electrically coupled neurons in GCaMP6s *Xenopus* tadpoles. First, we showed that loading 2 mM Ca^2+^ into neurons leads to an average fluorescence increase of 148.6% and an average decay halftime of 103 s. Ca^2+^ diffusion times across gap junctions have been reported previously. In coupled cochlear cells, it takes <1.5 s for elevated intracellular Ca^2+^ in one cell to lead to a detectable increase in Ca^2+^ in the other (Sun et al., 2005), whereas diffusion between coupled astrocytes and oligodendrocytes can take up to 155 s (Parys et al., 2010). Disparities between studies may reflect cell type, connexin composition, and methodological differences. In contrast to these studies investigating soma-somatically coupled cells, *Xenopus* dINs mostly have axo-axonal gap junctions (Li, Roberts and Soffe, 2009), which could be located hundreds of microns away from the soma and dendrites (Li and Soffe, 2019). More time and higher Ca^2+^ concentrations may be required for Ca^2+^ diffusion into the somas of coupled dINs.

We therefore increased the Ca^2+^ concentration in the intracellular solution to 10 mM. However, whole-cell recordings were extremely hard to maintain and displayed signs of rapid membrane resealing after breakthrough. Resealing was also observed in our 2 mM recordings, although these could be held in whole-cell mode for longer. Ca^2+^ is required for many plasma membrane repair mechanisms after injury (Steinhardt, Bi and Alderton, 1994; Cheng et al., 2015). During membrane damage, an influx of extracellular Ca^2+^ (∼2 mM) into the cytosol (∼100 nM Ca^2+^ at rest) signals cell damage (Clapham, 2007). Ca^2+^ sensors like synaptotagmin VII, annexins, and dysferlin detect rises in intracellular Ca^2+^ and promote various membrane repair mechanisms (Reddy, Caler and Andrews, 2001; Chakrabarti et al., 2003). Ca^2+^ chelators like EGTA and BAPTA can block membrane resealing by stabilizing free intracellular Ca^2+^, hence their inclusion in intracellular solutions for electrophysiology (Tsien, 1980). However, we excluded these from the intracellular solution to maintain high intracellular free Ca^2+^ and maximize diffusion. Consequently, Ca^2+^ loading likely triggered Ca^2+^-mediated membrane repair cascades, resulting in rapid loss of whole-cell recordings.

Instead of elevating Ca^2+^ concentration, we applied pharmacological blockers against *Xenopus* orthologs of SERCA2 proteins and NCX1.1. These blockers lowered maximum fluorescence increases in response to Ca^2+^ loading. The reason behind this is unknown; however, the addition of TTX and Cd^2+^ potentially reduced fluorescence increases from spontaneous cell activity and synaptic transmission, respectively. Importantly, they did not prolong fluorescence increases in the loaded cell, indicating the presence of active Ca^2+^ clearance mechanisms insensitive to these blockers. Many proteins help maintain Ca^2+^ homeostasis beside SERCAs and NCXs, including plasma membrane Ca^2+^ ATPases and Na^+^/Ca^2+^-K^+^ exchangers (Clapham, 2007). Adding more pharmacological blockers may sustain elevated cytoplasmic Ca^2+^ but may also pose greater risk to cell integrity. Instead, during stable recordings, we injected positive current steps to drive extra Ca^2+^ in the loaded cell. Although elevated fluorescence could be sustained in the target dIN throughout the injection period, no fluorescence increases were detected in neighboring cells, including dINs. This suggests that either more time is required for Ca^2+^ diffusion across axonal gap junctions or that Ca^2+^ diffused into coupled dINs was cleared too quickly or not detected by GCaMP6s. Indeed, detectable GCaMP6s fluorescence changes often require bursts of action potentials (Fig. 1*D*; Huang et al., 2021; Zhang et al., 2023). Single action potentials are estimated to increase free cytoplasmic Ca^2+^ by only 300–400 nM, lasting <2 ms (Helmchen et al., 1997; Sinha et al., 1997). More sensitive genetically encoded Ca^2+^ indicators may detect more transient, low concentration changes. This would, however, not overcome membrane resealing, which hinders introducing Ca^2+^ into the cell.

Gap junction permeability can also be regulated by cytoplasmic Ca^2+^. In many cases, high intracellular Ca^2+^ (nanomolar to micromolar ranges) gates or uncouples gap junctions (Délèze and Loewenstein, 1976; Dahl and Isenberg, 1980; Peracchia, 2004). However, some studies on Cx36 and Cx43 gap junctions showed elevated intracellular Ca^2+^ is required to open channels and increase conductance (Pereda et al., 1998; Del Corsso et al., 2012; Wang et al., 2012). Which connexins form gap junctions between dINs remains unknown. Since swimming activity triggers increases in Ca^2+^ and electrical coupling strength between dINs does not change immediately after swimming (Li et al, 2009), it is unlikely that high intracellular Ca^2+^ decreases gap junctional conductances in dINs. The distances between our recorded dINs (Figs. 4 and 5) ranged from 16 to 97 µm. Based on our previous empirical dataset (Li et al., 2009), we expected ∼10% coupling coefficients between these dINs, yet we saw little Ca^2+^ diffusion. The rapid Ca^2+^ clearance and membrane repair mechanisms suggested here preclude Ca^2+^ loading as a technically feasible method for revealing gap junction connections. Novel genetic lines expressing genetically encoded indicators of other ions (e.g., H^+^, K^+^, Cl^−^; Kang and Baker, 2016; Shen et al., 2019; Lodovichi et al., 2022) with lesser impacts on cell homeostasis and integrity may be potential paths to explore. These have been used in previously mentioned “generator-reporter” methods (Kang and Baker, 2016; Wu et al., 2019) but are currently restricted to models susceptible to extensive genetic manipulation. Small, biologically inert molecules remain the most promising avenue. Many cell labeling studies have replaced organic dyes with quantum dots: inorganic fluorophores with broader excitation and narrower emission spectra (Jaiswal et al., 2003; Resch-Genger et al., 2008). However, these were also found to regulate Cx43 gap junctional communication, depending on delivery mechanism and cell culture setup (Chang et al., 2009). The need remains for more widely applicable and accessible methods.

To conclude, this study attempted to explore Ca^2+^ loading using whole-cell recording and Ca^2+^ imaging as a method for revealing electrically coupled *Xenopus* tadpole neurons. We encountered two fundamental challenges: (1) Ca^2+^ does not persist long in the loaded cell, likely due to rapid homeostatic clearance, and (2) loading Ca^2+^ induces loss of whole-cell recordings due to membrane resealing. As a result, fluorescence coupling could not be revealed between neurons previously shown to be electrically coupled. We therefore conclude that imaging Ca^2+^ diffusion is not technically viable for revealing coupled *Xenopus* tadpole dINs.

## Synthesis

Reviewing Editor: Matthew Grubb, King's College London Decisions are customarily a result of the Reviewing Editor and the peer reviewers coming together and discussing their recommendations until a consensus is reached. When revisions are invited, a fact-based synthesis statement explaining their decision and outlining what is needed to prepare a revision will be listed below. The following reviewer(s) agreed to reveal their identity: Aalok Varma.

Synthesis:

Reviewers and Editors all agree that publishing negative results is important and appropriate for eNeuro. In this case, however, some key concerns need to be addressed before this negative result can be considered convincing and useful for the field. These are:

- A revision of the title to reflect the negative nature of the main finding

- A clear characterisation of the nature of the gap junction coupling which is under assessment. There are concerns that the failure of the method may be unique to this particular preparation, and not a generalisable limitation of the method itself. A revised manuscript needs to, at the very least, clarify the nature and strength of the gap junction coupling that the new method is attempting to assess. If this information is not available in the literature, a positive control is needed to demonstrate gap junction coupling, and its strength, between the neurons under study (e.g. paired recordings, as suggested by Reviewer 2). Even if such information on the expected nature and strength of the electrical coupling between these neurons is already published, a positive control would still considerably strengthen the main argument of the manuscript, so is greatly encouraged.

Please also ensure that you have addressed all of the Reviewers' individual comments below.

Reviewer 1:

Here the authors test whether infusing calcium into a given dIN of a GCaMP6 tadpole can be employed to identify the neurons gap junctionally coupled to this neuron.

It was found that loading a given neuron with calcium did indeed increase calcium concentration in the recorded neuron, but no noticeable increase in calcium was measured in any gap junction coupled neuron - so no increase in somatic calcium levels were observed beyond the filled neuron.

To rectify this, higher concentrations of calcium were loaded into the neuron, and this led to rapid membrane resealing. The authors tried various methods to enhance levels of free calcium in the neuron. They conclude that this approach is not useful for identifying gap junction coupled dINs.

This is an innovative idea for identifying gap junction coupled neurons. Unfortunately it does not appear useful for this purpose. Overall, this manuscript describes a failed method.

Additional Comments

The title does not reflect what the work shows. The title indicates that this is a new method for identifying electrically coupled neurons, but it is a failed method.

The authors fail to mention that gap junction coupled neurons in the tadpole optic tectum have been identified by filling one neuron with biocytin. Have the authors tried biocytin?

Reviewer 2:

In this manuscript, the authors attempt to use calcium imaging as a means of detecting gap junctional coupling between neurons in vivo in Xenopus larvae. Their idea was to load a target cell with a high concentration of calcium intracellularly, using patch electrodes, and monitor the spread of calcium to nearby, potentially electrically coupled cells, via calcium transients. The method was largely unsuccessful, for three reasons - (1) increase in GCaMP fluorescence wasn't persistent, (2) the high Ca2+ concentration used in the internal solution made whole-cell recordings unstable, and (3) no spread of GCaMP fluorescence was observed between neighbouring cells.

While the idea is, indeed, useful for animals with low genetic tractability, leveraging transgenic lines that are common and widely used, the experiments and some conclusions are not entirely convincing. The parts that are convincing are the following: (1) entering whole-cell mode with high internal Ca2+ leads to a large GCaMP6s transient, and (2) increased internal Ca2+ leads to membrane resealing and poor quality whole-cell recordings.

Major comments:

The title seems a bit misleading and should make it clearer that the method did not work.

What is the coupling coefficient of the dINs in question? Given that these gap junctions are axo-axonic, would you even detect sufficient calcium in the soma, which is what you're imaging? Do you have any back-of-the-envelope calculations to suggest that this is plausible in the preparation in question?

In continuation with the previous point, a true positive control experiment is needed here. Ideally, this would be a paired recording + imaging between dINs in your preparation. If you see a clear somatic gap-junctional coupling signal in the electrophysiology channel, but no corresponding calcium transient in the GCaMP channel, only then can you reasonably conclude that the method is the problem.

If your question is to simply identify the presence of gap-junctions between cells, I would suggest trying the gap-FRAP method, first published in 1986. That seems to be ideally suited to your project/problem, as it only requires a fluorescein-based dye, and no genetic lines.

Minor concerns:

Statistics in Fig 3B. Why was multiple correction applied? It doesn't seem appropriate, and moreover, it artificially makes two clearly different distributions appear not significantly different.

In Fig 4B and 5B, for completeness, can you also show the GCaMP traces obtained while recording using loose patch?
